# Trichobezoar

**DOI:** 10.5334/jbsr.2478

**Published:** 2021-05-21

**Authors:** Jonas De Melio, Thomas Debrouwere, Murielle Herman

**Affiliations:** 1 AZ Delta, BE

**Keywords:** Bezoar, Trichobezoar, Pediatric Radiology, Intragastric Mass, Trichophagia, Trichotillomania

## Abstract

**Teaching point:** A trichobezoar is a relatively rare entity that presents on imaging as a heterogeneous and multilayered mass molded by the stomach lumen.

## Case History

**Figure 1:**
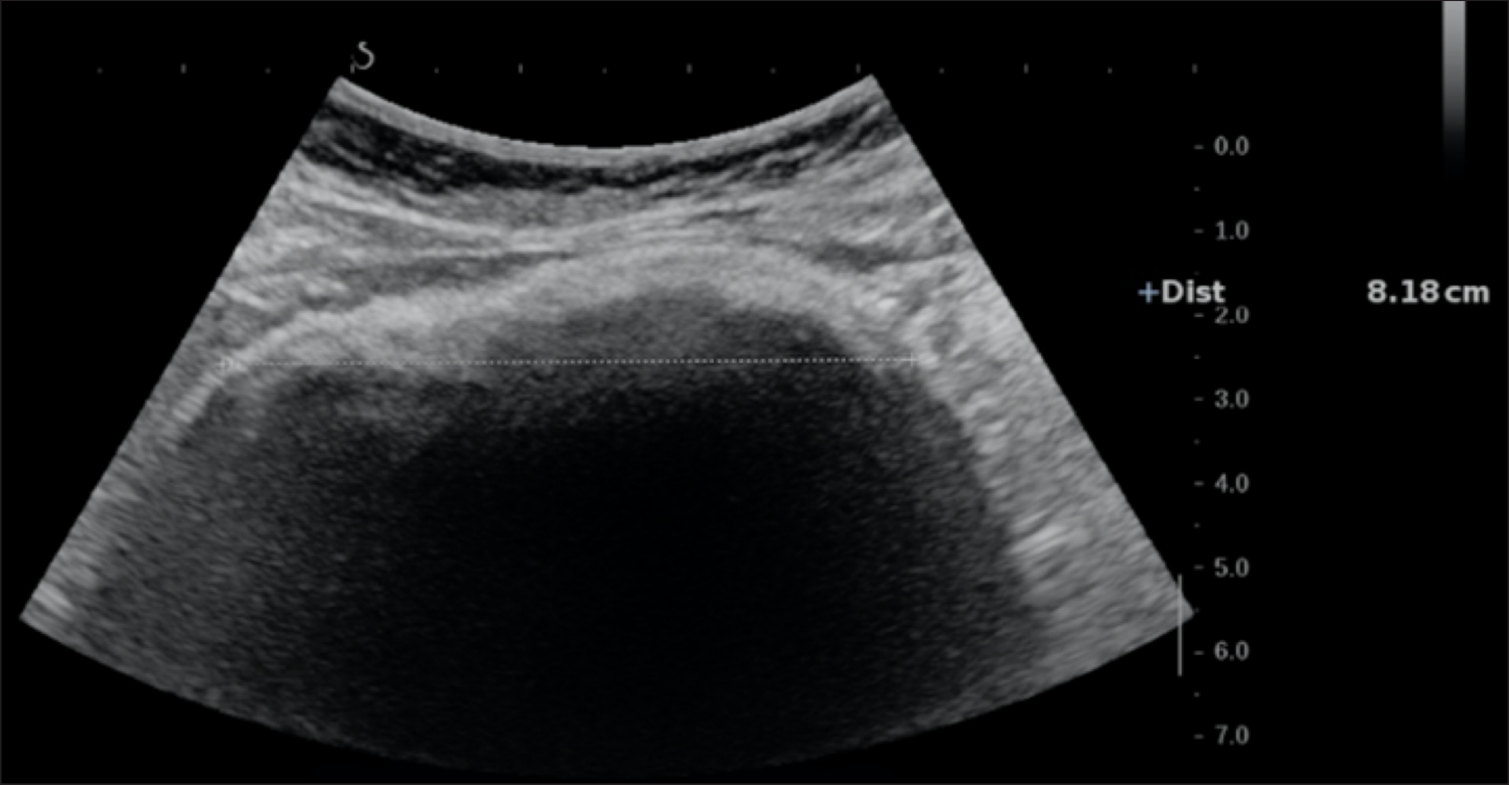


**Figure 2:**
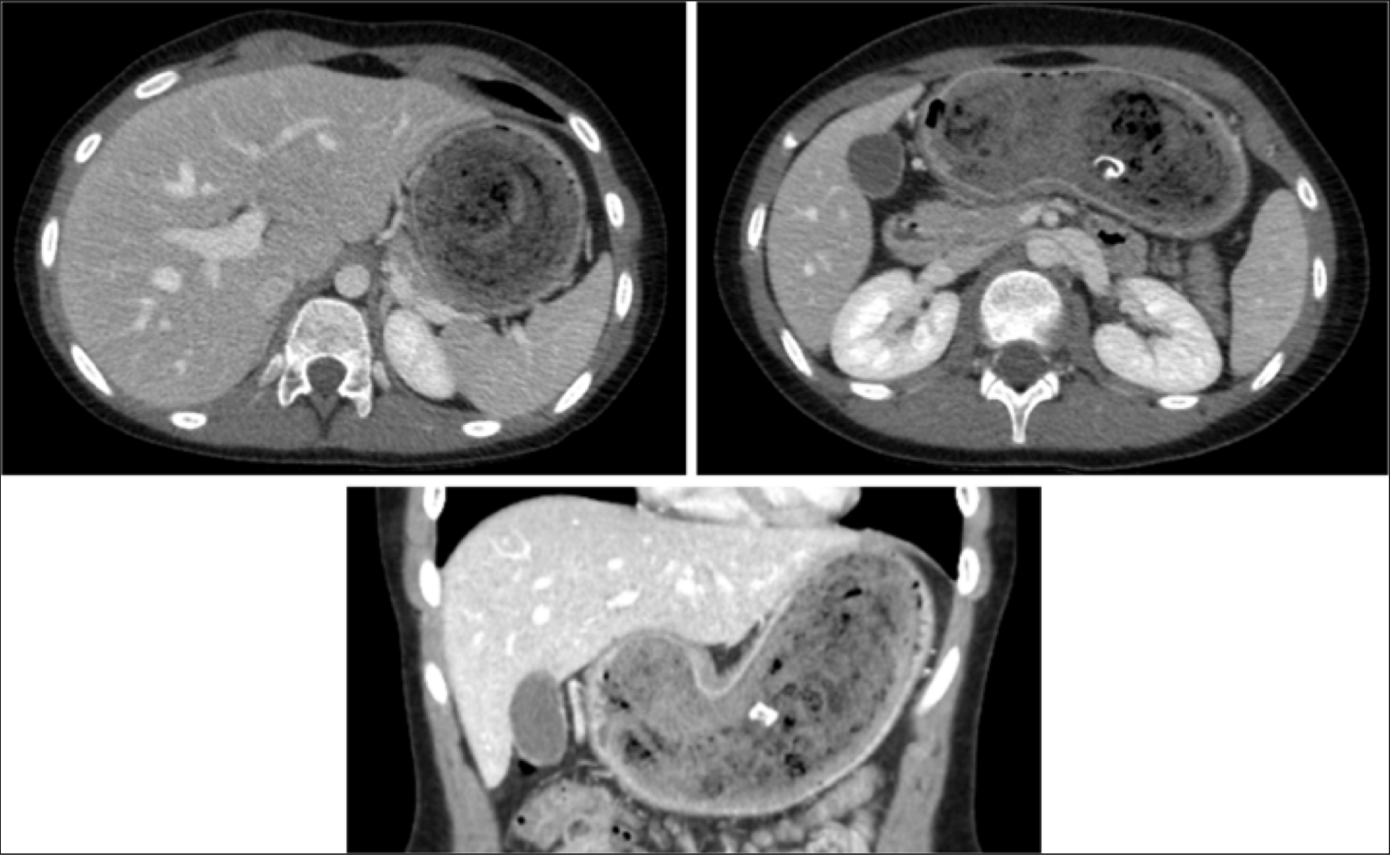


**Figure 3:**
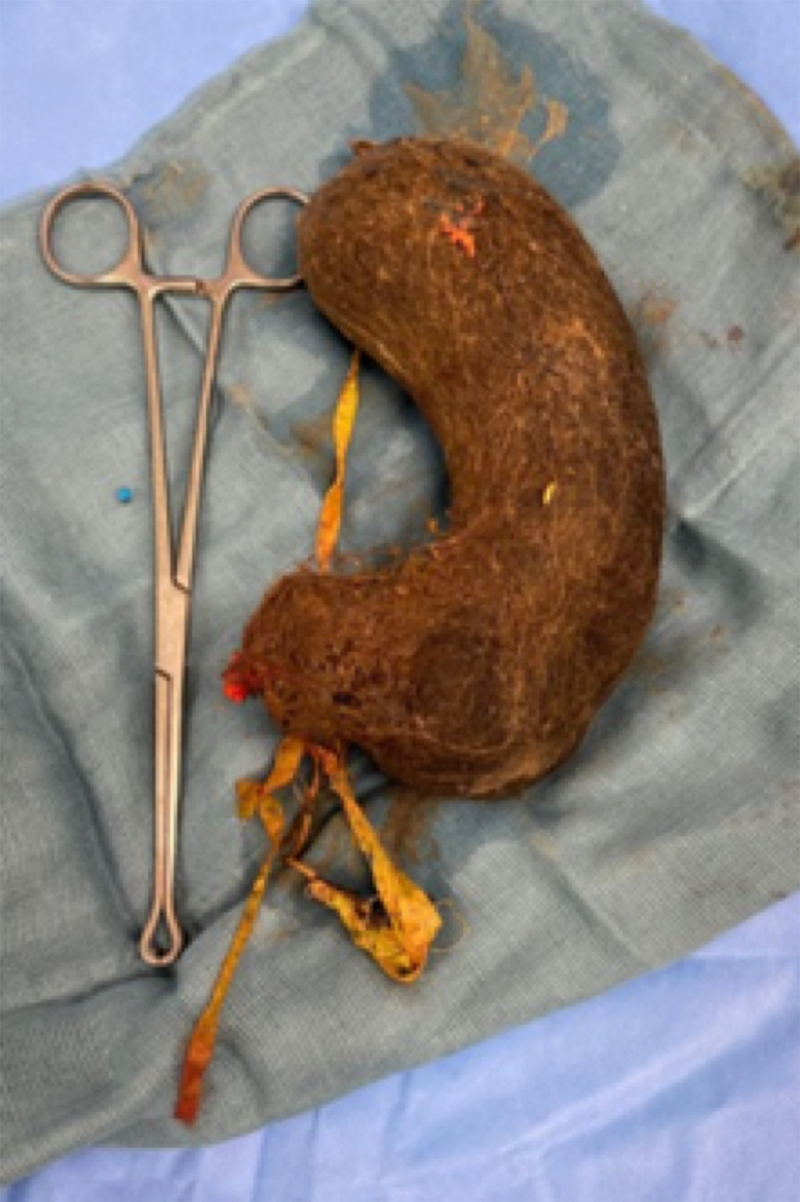


An 11-year-old girl presented to the gastroenterologist with complaints of burping, abdominal pain, and difficulties with eating for a couple of weeks. Clinical examination was suggestive of a mass in the left hypochondrium. An abdominal ultrasound was performed under fasting conditions, revealing a mass-like structure in the left hypochondrium with acoustic shadowing (***Figure 1***). A subsequent computed tomography (CT) revealed a multi-layered heterogenous mass, mixing hyperdensities, and gas bubbles, molded by a dilated stomach cavity (***Figure 2***). There was secondary dilatation of the stomach. The mass did not extend beyond the gastroduodenal junction. The CT diagnosis was unequivocally trichobezoar which was subsequently confirmed on gastroscopy that failed to remove it due to its dimensions. Laparotomy and gastrotomy were eventually performed to evacuate the trichobezoar (***Figure 3***). Psychological evaluation revealed that the child used trichophagia and trichotillomania to relieve stress resulting from social relations with peers.

## Comment

A bezoar is defined as an intraluminal conglomerate of difficult or non-digestible material that accumulates in the gastrointestinal (GI) tract. A bezoar can occur anywhere in the GI tract, but most of the bezoars are formed and found in the stomach. Rarely, there is a continuous extension of the mass beyond the gastroduodenal junction, the so-called Rapunzel syndrome. The classification of the bezoar depends on the material involved. The most common type is a phytobezoar, composed out of plant components. A trichobezoar, as in our case, is composed mainly out of hair. A trichobezoar is typically seen in young women. Some psychiatric disturbances, such as trichophagia and trichotillomania, are associated with a trichobezoar.

Symptoms of a bezoar can range from none to severe. The diagnosis can be made with endoscopy or imaging. Endoscopy has the advantage of direct visualization of the mass and the potential of direct therapeutic treatment, whereas the advantage of imaging lies in excluding other etiologies of the complaints. The imaging method of choice is CT, as plain X-rays and barium studies are insufficiently accurate. In the case of a phytobezoar, the typical appearance is an ovoid or round mass with gas bubbles and a mottled aspect, such as small bowel feces [1].
